# High-density lipoprotein sensor based on molecularly imprinted polymer

**DOI:** 10.1007/s00216-017-0442-3

**Published:** 2017-06-29

**Authors:** Suticha Chunta, Roongnapa Suedee, Peter A. Lieberzeit

**Affiliations:** 1 University of Vienna, Faculty for Chemistry, Department of Physical Chemistry, Währinger Straße 42, 1090 Vienna, Austria; 2 Department of Pharmaceutical Chemistry, Faculty of Pharmaceutical Sciences, Prince of Songkla University, Hat Yai, Songkhla 90112 Thailand

**Keywords:** High-density lipoprotein, Molecularly imprinted polymer, Quartz crystal microbalance, Lipoprotein sensor

## Abstract

Decreased blood level of high-density lipoprotein (HDL) is one of the essential criteria in diagnosing metabolic syndrome associated with the development of atherosclerosis and coronary heart disease. Herein, we report the synthesis of a molecularly imprinted polymer (MIP) that selectively binds HDL, namely, HDL-MIP, and thus serves as an artificial, biomimetic sensor layer. The optimized polymer contains methacrylic acid and *N*-vinylpyrrolidone in the ratio of 2:3, cross-linked with ethylene glycol dimethacrylate. On 10 MHz dual electrode quartz crystal microbalances (QCM), such HDL-MIP revealed dynamic detection range toward HDL standards in the clinically relevant ranges of 2–250 mg/dL HDL cholesterol (HDL-C) in 10 mM phosphate-buffered saline (PBS, pH = 7.4) without significant interference: low-density lipoprotein (LDL) yields 5% of the HDL signal, and both very-low-density lipoprotein (VLDL) and human serum albumin (HSA) yield 0%. The sensor reveals recovery rates between 94 and 104% at 95% confidence interval with precision of 2.3–7.7% and shows appreciable correlation (*R* ^2^ = 0.97) with enzymatic colorimetric assay, the standard in clinical tests. In contrast to the latter, it achieves rapid results (10 min) during one-step analysis without the need for sample preparation.

**Graphical Abstract:**
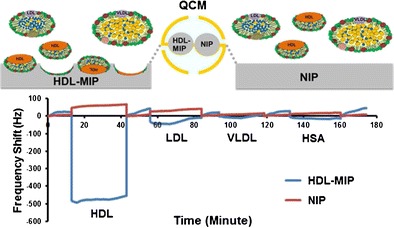
ᅟ

ᅟ

## Introduction

High-density lipoprotein (HDL) plays an essential role as antiatherogenic marker in the reverse cholesterol transport pathway [1]. It does so by inhibiting oxidation of low-density lipoprotein (LDL) and hence preventing formation of oxidized LDL, which is a crucial atherogenic factor [2]. Furthermore, HDL shows anti-inflammatory activity by inhibiting the production of inflammatory cytokines. Those induce expression of vascular cells and intracellular adhesion molecules on the coronary vascular [3]. Therefore, decreased blood level of HDL means increased risk for developing metabolic syndrome and atherosclerosis and finally coronary heart disease (CHD) [4, 5]. Current clinical analysis approximates the serum concentration of actual HDL particles by determining the amount of cholesterol bound to HDL (HDL-C) in the serum [6] due to difficulties in measuring actual HDL particles in blood using any standard methods. Concentrations below 40 mg/dL HDL-C are related to high risk of CHD incidence, whereas values equal to or higher than 60 mg/dL indicate protection against CHD [5]. In clinical analysis, HDL-C is analyzed by an enzymatic colorimetric assay that utilizes cholesterol esterase, cholesterol oxidase, and peroxidase coupled with UV-Vis photometry [7]. This method requires sample pretreatment by precipitating all other serum proteins using reagents such as heparin [8], 50,000 Da dextran sulfate, phosphotungstic acid, polyethylene glycol [9], or dextran sulfate-coated iron particles [10] in the presence of divalent cations (e.g., Mg^2+^, Mn^2+^) [8, 9]. After centrifugation or magnetic separation, only HDL remains in the supernatant. However, increased blood levels of triglycerides or triglyceride-rich lipoproteins (e.g., very-low-density lipoprotein—VLDL) can interfere with precipitation and prevent sedimentation of aggregates. Therefore, supernatants may be contaminated with other lipoproteins leading to systematically too high results for HDL-C [7]. Assay selectivity for HDL can be improved by adding polyethylene glycol beads coated with specific antibodies binding to serum apolipoprotein (Apo) B or C. Apo-B and Apo-C are present in several lipoproteins, namely, chylomicron, VLDL, intermediate-density lipoprotein (IDL), and LDL [10]. The immune reaction hence eases precipitation. In a different approach, one can use a polyanion and synthetic polymer agents to block non-HDL lipoproteins before adding the colorimetric cholesterol reagents to determine HDL-C [11]. Although the enzymatic HDL-C assay is inherently highly selective to cholesterol, it is limited by the abovementioned selectivity issues, as well as stability and high cost of antibodies and enzymes. Furthermore, it turned out that increased serum concentrations of triglycerides, bilirubin, ascorbic acid, free hemoglobin, and gamma-globulin, respectively, interfere [7].

As a consequence of such limitations, artificial recognition elements attract increasing interest in sensing. Molecularly imprinted polymers (MIPs) represent such biomimetic receptors showing appreciable selectivity, storage stability, resistance against biofouling, and reusability [12]. MIPs contain functionalized cavities whose exact shape and surface chemistry is determined by self-organization between a growing polymer matrix and a template species, usually the target analyte [13]. To date, a wide range of MIPs has been published covering small molecules as well as whole cells [14, 15]. Other bioanalytical applications of MIP-based sensors include detecting bio(macro)molecules, such as sugars [16], cholesterol [17], phospholipids [18], and proteins [19]. Some MIPs have already been applied in clinical diagnosis and therapeutic monitoring, for instance to determine the concentration of human serum albumin (HSA) in serum [20], nicotine [21] or creatinine [22] in urine, and also in ABO blood group typing in whole blood [23]. Recently, we reported the successful sensing of LDL with MIP-based quartz crystal microbalance (QCM) sensors (LDL-MIP sensor) directly in serum [24]. Herein, we report the design of corresponding HDL-MIP sensors. The challenges for that were twofold: firstly, detection limits in the case of HDL have to be lower due to the lower clinically relevant threshold concentration (lower than 40 mg/dL for HDL-C, higher than 129 mg/dL for LDL-C). Secondly, LDL and HDL are both composed of similar types of components, namely, triglycerides and cholesteryl esters within the lipoprotein particle surrounded by a layer of amphipathic phospholipid, free cholesterol, and apolipoprotein. LDL and HDL differ by the ratios of these constituents and hence also slightly in diameter (21.5 ± 6.5 nm for HDL, 28.9 ± 9.2 nm for LDL) [25].

## Materials and methods

### Chemicals

Methacrylic acid (MAA), *N*-vinylpyrrolidone (VP), dimethyl sulfoxide (DMSO), potassium chloride (KCl), and agarose powder were purchased from VWR International (Vienna, Austria); *N,N*′-(1,2-dihydroxyethylene)bisacrylamide (DHEBA), 2,2′-azobis(isobutyronitrile) (AIBN), sodium bromide (NaBr), and Sudan Black B were from Sigma-Aldrich (Steinheim, Germany). Tris(hydroxymethyl)-aminomethane (Tris), ethylenediamine tetraacetic acid (EDTA), calcium chloride (CaCl_2_), magnesium chloride (MgCl_2_), urea (CH_4_N_2_O), and (D+)-glucose monohydrate were purchased from Merck (Darmstadt, Germany). Sodium chloride (NaCl) was obtained from Applichem (Darmstadt, Germany). Acetic acid was purchased from Carl Roth (Karlsruhe, Germany). 4-(2-Hydroxyethyl)piperazine-1-ethanesulfonic acid (HEPES) was obtained from Alfa Aesar (Karlsruhe, Germany). HSA was purchased from Millipore (MA, USA). Brilliant gold paste (gold colloid, 12% gold content) was purchased from Heraeus, Germany. All reagents were of analytical or highest synthetic grade commercially available.

### Lipoprotein isolation

Human sera were taken from a volunteer at the Faculty of Medical Technology, Prince of Songkla University. Gradient density ultracentrifugation was utilized to isolate lipoprotein classes—VLDL, LDL, and HDL—as described in the following paragraph using a Beckman Coulter Optima L-100 XP ultracentrifuge with a fixed angle rotor type 100 Ti 100,000 rpm [24] as shown in Table 1. All centrifugations were carried out at 80,000 rpm at 4 °C. Briefly, the first step comprised of layering 2 mL of 0.195 M NaCl solution (*ρ* = 1.006 g/mL) on top of 4 mL human serum in a centrifuge tube. Centrifugation for 10 h yielded the VLDL fraction in the top layer. The bottom layer containing LDL, HDL, and other serum proteins was transferred to a new centrifuge tube followed by layering 2 mL of a solution containing 0.195 M NaCl and 2.44 M NaBr (*ρ* = 1.063 g/mL). After centrifuging for 14 h, the LDL fraction could be collected in the top layer. The bottom layer containing HDL and other serum proteins was transferred to another tube filled with 2 mL of a solution containing 0.195 M NaCl and 7.65 M NaBr (*ρ* = 1.478 g/mL). After mixing and centrifugation for 10 h, the top layer comprising HDL was collected [24]. Each fraction was characterized by 0.5% agarose gel electrophoresis on Bio-Rad subcell GT electrophoresis systems. The procedures for electrophoresis and determining lipid content in the gels by staining with 0.4% Sudan Black B were similar to those described previously [24]. Cholesterol concentrations in each fraction such as VLDL-C, LDL-C, and HDL-C standard solutions were determined on a Roche Hitachi 917 chemistry autoanalyzer via homogeneous enzymatic colorimetry. As previously mentioned, actual lipoprotein particle concentrations in blood are difficult to determine by current instrumental techniques. Hence, clinical reference ranges define HDL content in terms of HDL-C concentration, i.e., via determining the concentration of cholesterol bound to HDL. Therefore, all HDL-MIP sensor signals are calibrated against HDL-C rather than “actual” HDL concentration, making the results compatible to diagnostic methods.

**Table 1:** Conditions of ultracentrifugation for serum lipoprotein isolation

Ultracentrifuge rotor type 100 Ti, 80,000 rpm at 4 °C			Fractions obtained in the top layer	Lipoprotein density (g/mL)
Type of salt	Medium density (g/mL)	Time (h)		
NaCl	1.006	10	VLDL	0.94–1.006
NaCl/NaBr	1.063	14	LDL	1.006–1.063
NaCl/NaBr	1.478	10	HDL	1.063–1.21

### QCM transducer fabrication

Ten megahertz (MHz) QCM were fabricated by screen printing dual gold-electrode configuration onto commercially available AT-cut quartz plates (168 μm thick, 13.8 mm diameter; Great Microtama Industries, Surabaya, Indonesia) with a brilliant gold paste (Heraeus; 12%). Then, they were baked in the oven at 400 °C for 4 h. After measuring the resonance frequency and damping with an Agilent 8712ET network analyzer, QCM transducers with less than −5 dB damping were selected for further use [24].

### Synthesis of HDL-MIP

**Fig. 1:**
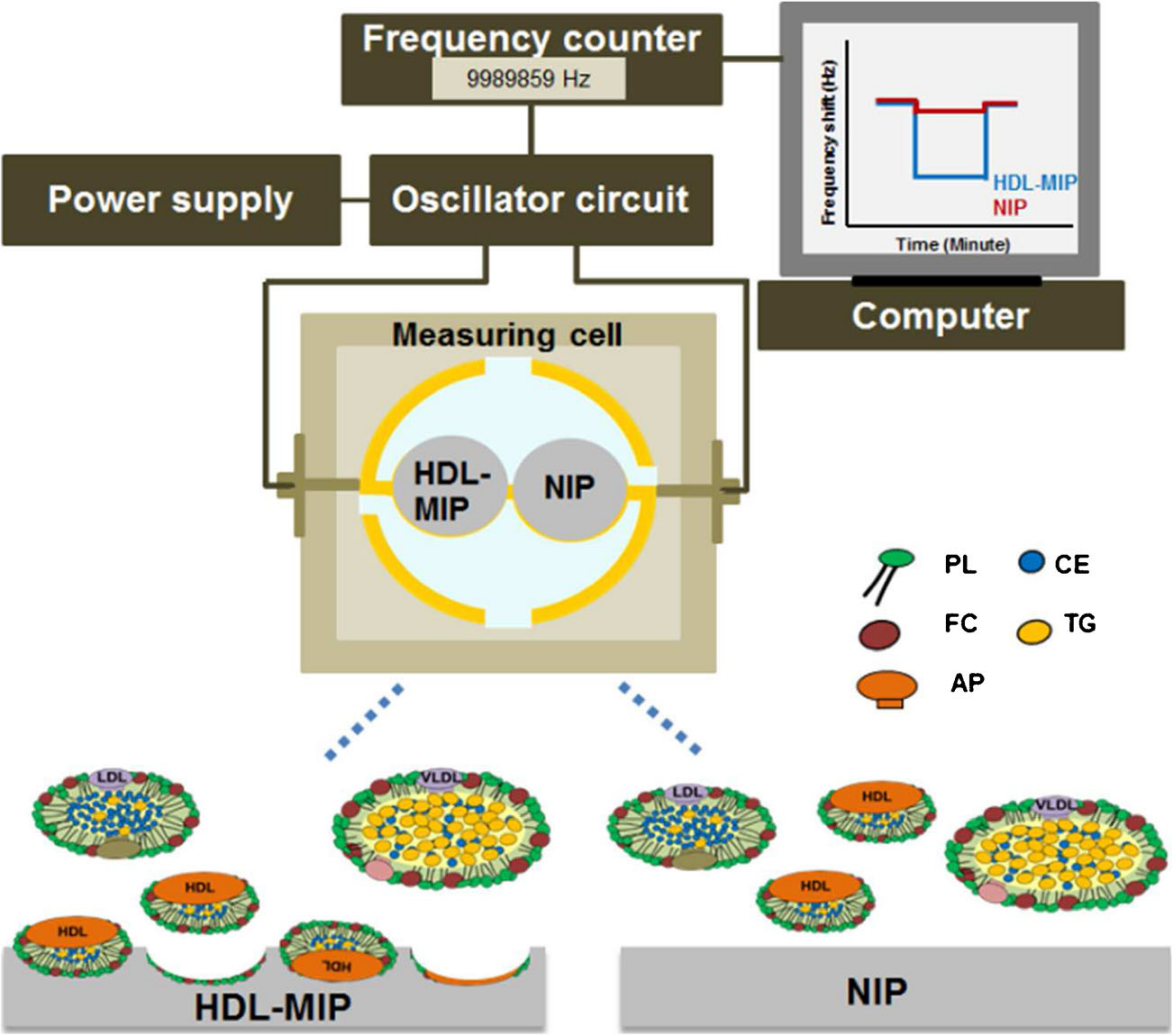
Schematic diagram of the QCM setup and mode of action of HDL-MIP and NIP reference (*HDL-MIP* high-density lipoprotein-molecularly imprinted polymer, *NIP* nonimprinted polymer, *PL* phospholipid, *TG* triglyceride, *FC* free cholesterol, *CE* cholesterol ester, *AP* apolipoprotein)

Imprinting protocol and copolymer conditions followed the procedure used for the synthesis of our previously published sensor of LDL-MIP [24]. Polymer optimization included varying the ratio of functional monomers MAA and VP (1:4, 2:3, and 3:2) and keeping the amount of cross-linker (DHEBA) constant at 70% (*w*/*w*). Briefly, all polymer systems consisted of 15 mg of binary monomer mixture, 35 mg of DHEBA, and 2.4 mg of the initiator AIBN dissolved in 300 μL DMSO. These solutions were prepolymerized under filtered UV lamps VL-215.LM at 365 nm, 15 W, for 20 min until just prior to reaching the gel point. Afterward, 5 μL of this oligomer solution was drop-coated onto the entire sample side of a dual electrode QCM and spun off at 3000 rpm for 2 min to obtain a thin layer of prepolymer. Subsequently, 5 μL of HDL standard corresponding to 400 mg/dL HDL-C was dropped directly onto the prepolymer layer above one of the electrodes, spun off for a few seconds, and covered with a clean glass slide to yield the HDL-MIP. The polymer on the reference electrode side was not exposed to HDL to yield the nonimprinted polymer (NIP). Then, the polymer layer on QCM was completely polymerized at 50 °C for 12 h. Templates were removed by stirring in 10% aqueous solution of acetic acid followed by 0.1% sodium dodecyl sulfate (SDS) solution and deionized water for 20 min each. Figure 1 summarizes the sensor setup and the different functional parts.

### Polymer characterization

Topographic images of HDL-MIP and NIP were recorded by atomic force microscopy (AFM) in contact mode. AFM was operated in air using a Bruker Instruments NanoScope VIII with a silicon nitride cantilever (ScanAsyst-air) at 0.5 Hz scan rate.

### QCM measurements

QCM were mounted in a custom-made cell connected to the oscillator circuit following a previously described protocol [26], which is also shown in Fig. 1. A typical measurement comprised of several steps: first, 180 μL of 10 mM phosphate-buffered saline (PBS, pH = 7.4) was injected into the measuring cell to obtain baseline signal. Afterward, the cell was flushed with 180 μL standard HDL-C solutions (3.12–350 mg/dL) in 10 mM PBS, respectively. All measurements were carried out in stopped flow until the signal reached its equilibrium state. Afterward, we washed the cell with 10% aqueous solution of acetic acid, followed by 0.1% SDS solution, and finally deionized water (10 min each at a flow rate of 0.46 mL/min) [26].

### HDL-MIP sensor characterization

HDL-MIP sensors were characterized in terms of limit of detection, accuracy, precision, analytical sensitivity, and selectivity. Accuracy of HDL-MIP sensors was examined by recovery tests at clinically “low” and “normal” HDL-C concentrations. Test samples were prepared by adding different volumes of standard HDL-C solution at a concentration of 300 mg/dL (namely, 10, 40, and 80 μL) to 200 μL of 20 mg/dL HDL-C solution to reach final concentrations at 33.33, 66.66, and 100 mg/dL HDL-C, respectively. The corresponding frequency shifts were compared to the values expected from calibration of the sensor. For testing reproducibility, the sensor responses of HDL-C standard solutions containing 20, 50, and 100 mg/dL, respectively, were recorded three times each. For determining selectivity, the HDL-MIP sensor was exposed to standard solutions of possible interfering species at high concentrations that can be found in human serum, namely, 150 mg/dL LDL-C, 80 mg/dL VLDL-C, and 1000 mg/dL HSA, respectively.

### Clinical sample measurement

Different volumes of standard HDL-C solution at a concentration of 385 mg/dL (namely, 10, 30, 50, and 60 μL) were spiked to different volumes of human serum with known HDL-C concentration *c* = 63 mg/dL (namely, 390, 370, 350, and 340 μL) to reach a final volume of 400 μL. All sera were diluted by mixing 1 part serum and 1 part PBS to reduce matrix effects prior to measurement [24].

As we did not have access to clinical samples with low HDL-C concentrations, those standards were prepared in “artificial serum” (AS). It contained 0.1% HSA, 4.5 mM KCl, 5 mM CaCl_2_, 4.7 mM (D+)-glucose monohydrate, 2.5 mM urea, 145 mM NaCl, and 1.6 mM MgCl_2_ in 200 mM HEPES buffer (pH = 7.4) [27]. Then, HDL-free “artificial” serum was prepared by adding 100 mg/dL of LDL-C standard and 20 mg/dL VLDL-C standard. Different volumes of a standard HDL-C at a concentration of 385 mg/dL (namely, 5, 10, 20, 30, and 40 μL) were spiked to different volumes of HDL-free artificial serum (namely, 395, 390, 380, 370, and 360 μL) to reach a final volume of 400 μL at the concentrations of 4.8, 9.6, 19.3, 28.9, and 38.5 mg/dL HDL-C, respectively. Two types of assay matrixes, namely, 10 mM PBS and diluted HDL-free artificial serum (1 part HDL-free artificial serum plus 1 part 10 mM PBS), were utilized to achieve baseline signal. All spiked sera were diluted with 10 mM PBS by 1:2 prior to sensor measurements.

## Results and discussion

### Optimizing HDL-MIP synthesis

**Fig. 2:**
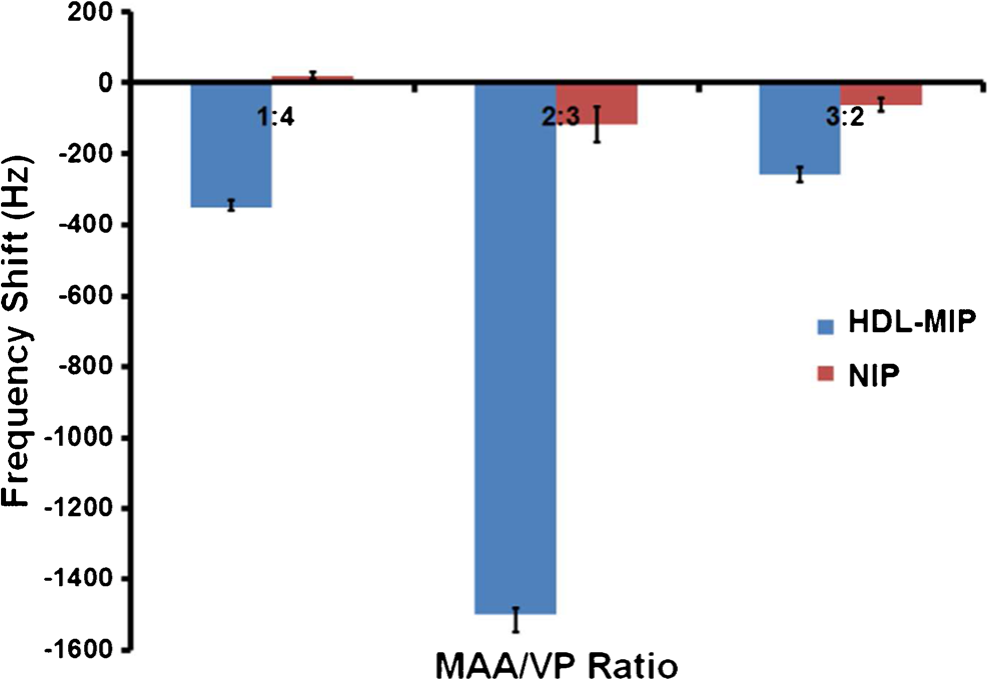
QCM frequency responses of HDL-MIP and NIP-coated electrodes toward a standard HDL-C solution at the concentration of 200 mg/dL at different ratios of MAA/VP

The surface of HDL particles is rather similar to LDL: it comprises a hydrophilic complex of phospholipids, free cholesterol, and apolipoprotein. Hence, we used the successful LDL-MIP [24] as a starting point for HDL-MIP synthesis and mixed monomers MAA/VP in a ratio of 3:2 (*w*/*w*). The most right-hand data in Fig. 2 shows the QCM frequency responses of both HDL-MIP and NIP-coated electrodes when exposing them to a standard HDL solution at a concentration of 200 mg/dL in PBS: this leads to a decreasing frequency by −258 Hz on the HDL-MIP side and −60 Hz on the NIP side, corresponding to −198 Hz mass effect. Obviously, the polymer shows some inherent affinity to HDL, which leads to sensor responses on the NIP and is up to some extent desirable to achieve MIP with high affinity [28]. Still the HDL-MIP leads to four times higher sensor responses, thus indicating successful imprinting. However, the previously published, corresponding LDL-MIP sensor revealed Δ*f* = −2850 Hz for LDL-MIP and Δ*f* = −273 Hz for NIP [24], so the first HDL-C responses shown in Fig. 2 are comparably low. One reason for this is that the surfaces of LDL and HDL are different: the HDL surface contains a higher amount of apolipoprotein, namely, 50%, compared to 25% in LDL [29]. Besides, the surface potential of HDL is more negative than that of LDL, namely, −10.5 to −12.5 mV vs. −4.5 to −7.0 mV. Therefore, we varied the MAA/VP ratio in the functional monomer by increasing the amount of VP to 1:4 and 2:3. Figure 2 compares both HDL-MIP and NIP signals for all three ratios: in the case of MAA/VP (1:4), the HDL-MIP yields slightly larger signal than before (−350 vs. −258 Hz), which indicates improved HDL binding. The NIP gives rise to slightly positive frequency shifts in the range of +20 Hz. Such anti-Sauerbrey behavior has previously been observed for interactions between biospecies and very smooth polymer surfaces [30, 31]. In the case of MAA/VP (2:3), both the HDL-MIP and NIP respond with the largest frequency shifts, namely, −1500 Hz for the HDL-MIP and −115 Hz for the NIP. This means increasing both by factors of six and two, respectively, and clearly demonstrates increased affinity of the material. By reducing the amount of methacrylic acid, the overall charge of the polymer is shifted further toward positive values, which is in line with the surface charges mentioned earlier. Hence, all further experiments utilized MAA/VP ratio at 2:3.

### Polymer characterization

**Fig. 3:**
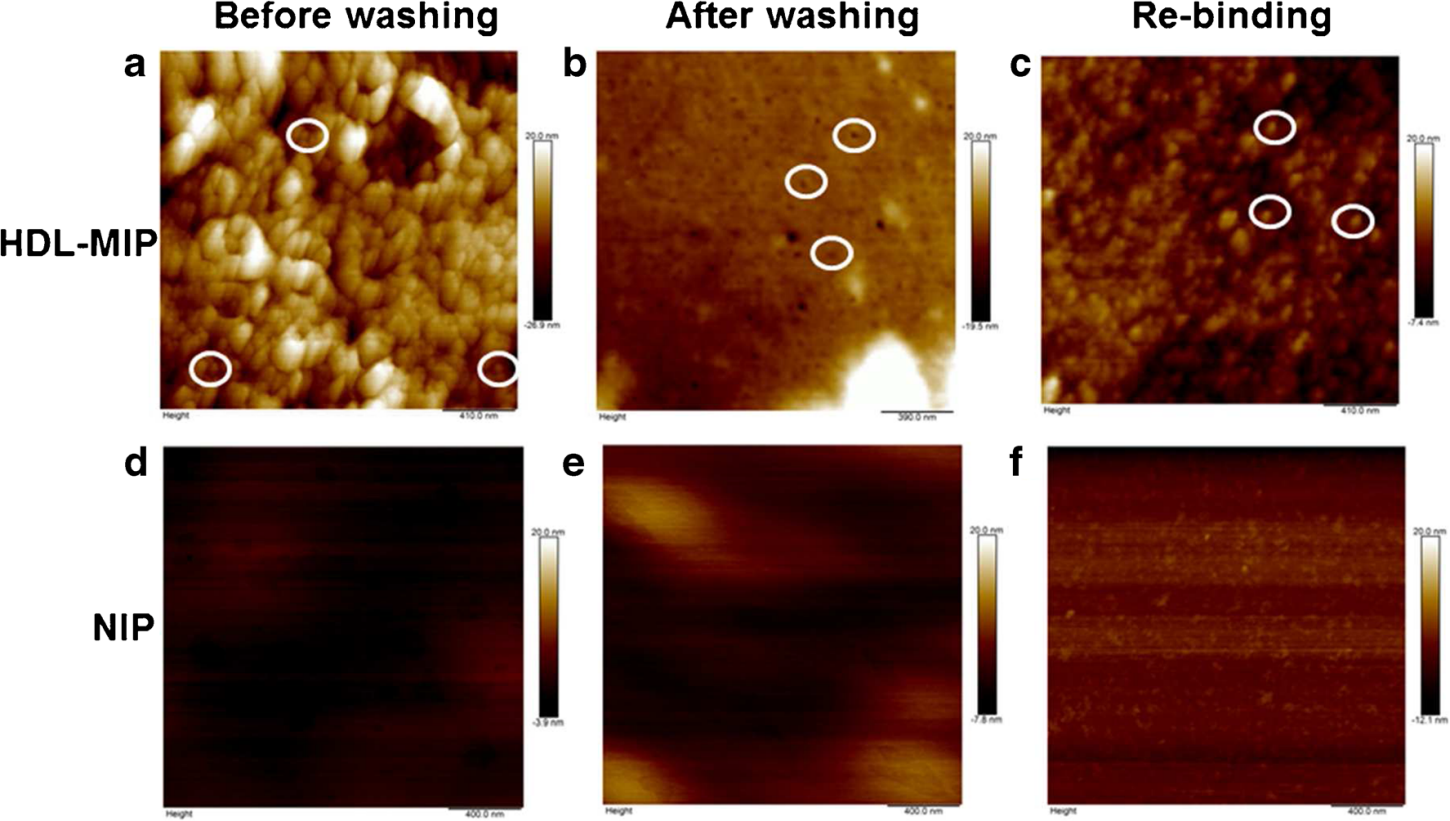
AFM images of polymer before removing the template (**a** HDL-MIP and **d** NIP), after removing the template (**b** HDL-MIP and **e** NIP), and rebinding with HDL-C at the concentration of 200 mg/dL (**c** HDL-MIP and **f** NIP)

Figure 3 shows AFM images in air of six different surfaces of the HDL-MIP and the NIP, namely, HDL-MIP before (Fig. 3a) and after (Fig. 3b) removing the template and HDL-MIP after rebinding HDL (Fig. 3c) as well as the corresponding NIP (Fig. 3d–f). HDL-MIP before removing the template (Fig. 3a) shows a large number of spherical structures with an average diameter of 42 ± 20 nm and a height of 1.9 ± 1.2 nm (*n* = 20). After washing, the HDL-MIP surface (Fig. 3b) reveals circular cavities that are on average 46 ± 15 nm across and 1.2 ± 0.8 nm deep (*n* = 17). These two diameters correspond well to one another. However, both the average diameters of HDL and HDL-MIP cavities are larger than the typical shape of HDL with a diameter and a height of 21.5 ± 6.5 and 4.1 ± 0.9 nm in liquid and 23.7 ± 6.9 nm across and 2.2 ± 0.4 nm high in air [25]. There are two reasons for this apparent increase in diameter: firstly, HDL in this case lies on a surface, which increases diameter and decreases thickness. Secondly, and more importantly, individual HDL particles may aggregate: HDL-MIP precursor solutions contain DMSO, which is known to stimulate protein aggregation. After exposing the layers to a standard HDL-C solution at a concentration of 200 mg/dL, HDL-MIP displays discrete circular particles representing reuptake of HDL on the HDL-MIP surface (Fig. 3c), whereas all NIP surfaces (Fig. 3d–f) lack those features. This strongly supports the successful synthesis of HDL-MIP both in terms of structure and functionality.

### HDL-MIP sensor characterization

#### Dose-response studies

**Fig. 4:**
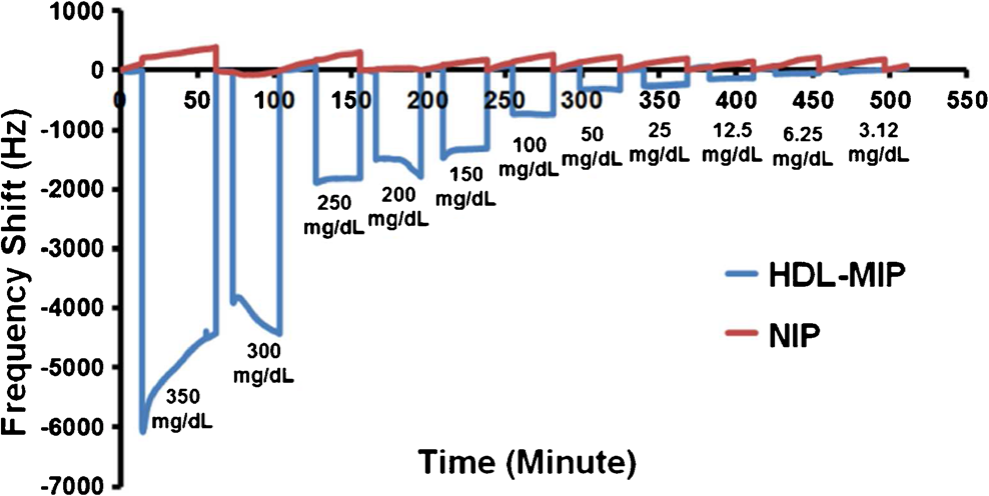
QCM responses toward different concentrations of standard HDL-C in 10 mM PBS

**Fig. 5:**
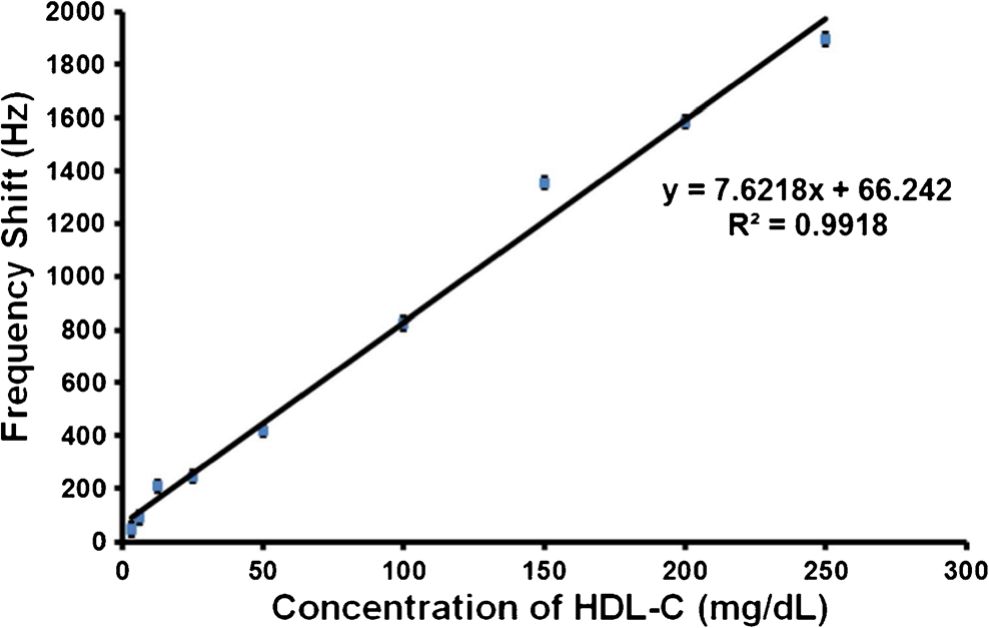
Linear response characteristic toward different concentrations of standard HDL-C in 10 mM PBS

Figure 4 shows the QCM sensor responses of HDL-MIP and NIP, respectively, toward different standards ranging between 3.12 and 350 mg/dL HDL-C. Sensor signals were read out after reaching the respective equilibrium frequency shift. In case of signal drift at 150, 300, and 350 mg/dL, respectively, the final frequency values of the respective signals were used. For HDL-C at 200 mg/dL, we used the constant value before further onset of signal drift. HDL-MIP clearly yields substantial mass responses in the range of −48 to −4418 Hz. In contrast to this, corresponding NIP gives rise to only slight frequency shifts that do not depend on concentration in a range of −5 to −274 Hz. All sensor responses turned out fully reversible. Obviously, the two standards at the upper end of the concentration range already lead to signal saturation on the sensors. Figure 5 shows the corresponding sensor characteristic and regression analysis: the HDL-MIP leads to linear sensor response with a correlation coefficient (*R* ^2^) of 0.9918. The limit of detection (LOD) and quantification (LOQ) of the HDL-MIP sensor were calculated from the signal to noise ratio, corresponding to 3 and 10 times the noise level, respectively. At 3.12 mg/dL HDL-C, the HDL-MIP sensor response is −48 Hz at a noise signal of 10 Hz leading to LOD and LOQ of 2 and 6.5 mg/dL HDL-C, respectively. Therefore, this sensor dynamically responds to HDL-C concentrations between 2 and 250 mg/dL, which corresponds to the required clinical concentration range: a cross-sectional study of 181 patients receiving medical care in a community hospital revealed a range of HDL-C at 23–94 mg/dL [32]. Concentrations indicating increased risk of CHD or metabolic syndrome status are below 40 and 50 mg/dL HDL-C for males and females, respectively, while normal ranges are 40–50 mg/dL HDL-C in males and 50–59 mg/dL in females, and protective status is above 60 mg/dL HDL-C for both males and females [33].

Table 2 compares these data with literature studies reporting HDL-C measurements using QCM or fiber-optic-based HDL-C immunosensors [34, 35] and conventional homogeneous enzymatic colorimetric assay [7]. Obviously, the HDL-MIP sensors presented here show both lower limit of detection and higher dynamic range, than the other methods, combined with shorter detection time, namely, 10 min. Moreover, 28% of a total of 84 fasting sera required dilution before QCM-based immunosensor measurement due to the limited dynamic range at concentrations higher than 58 mg/dL [34].

**Table 2:** Methods for HDL-C determination

Methods for HDL-C determination	Detection range (mg/dL)	Detection time (min)
QCM-based HDL-MIP sensor	2–250	10
Homogeneous enzymatic colorimetric assay	3–200	10–20
QCM-based HDL immunosensor	26–58	60
Fiber-optic-based HDL immunosensor	40–230	25

#### Accuracy

**Fig. 6:**
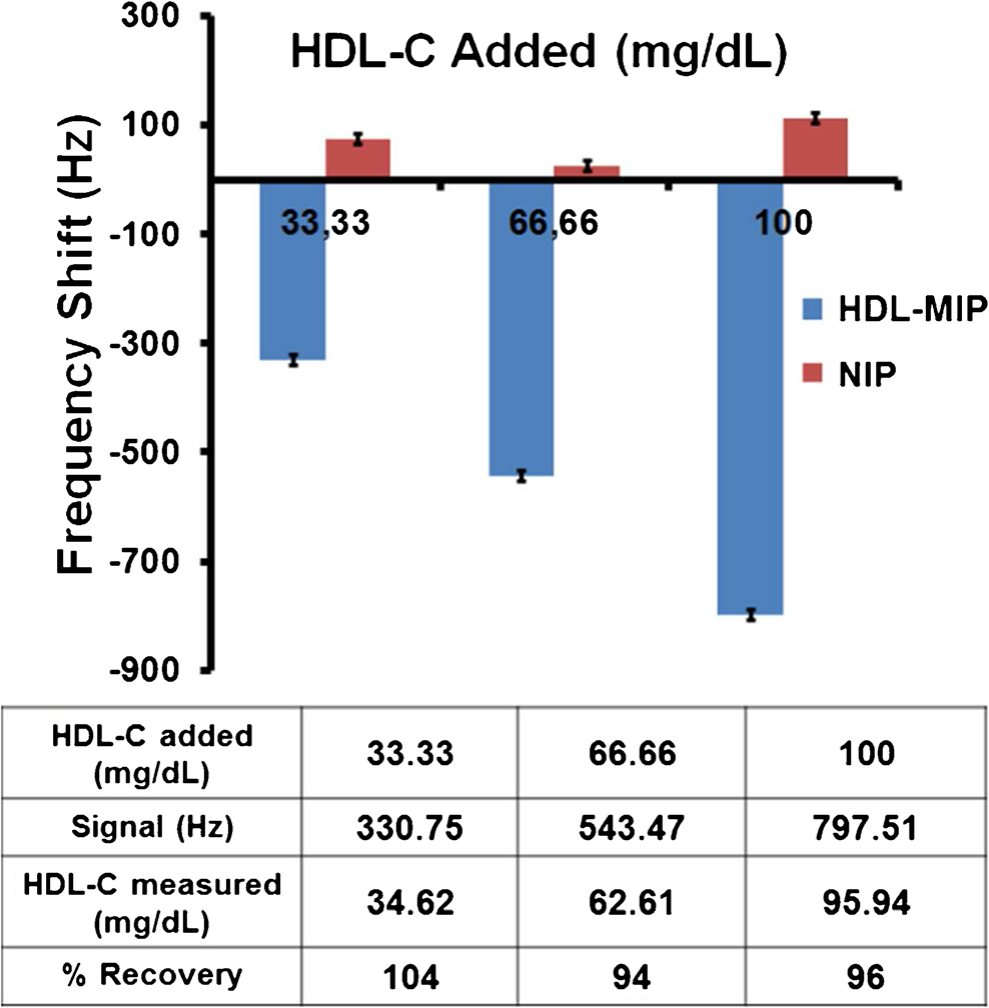
Recovery rates of HDL-MIP sensor

Figure 6 displays the recovery rates of the HDL-MIP sensor, which were calculated by comparing the concentrations obtained from sensor responses to the expected concentrations of the spiked HDL-C samples. They are 104, 94, and 96% at HDL-C at concentrations of 33.33, 66.66, and 100 mg/dL, respectively. Most published reports on the accuracy of homogeneous assays result in 95.5–97.8% accuracy when standard materials are used [7]. The National Cholesterol Education Program (NCEP) criteria for HDL-C measurements state that recovery rates should be between 95 and 105% to be acceptable; our HDL-MIP-based QCM system hence leads to acceptable results within the clinically relevant working range.

#### Reproducibility

Repeatability of our sensor assay at standard HDL-C solution at concentrations of 20, 50, and 100 mg/dL reveals coefficient of variation (CV) of 7.7, 2.3, and 3.4%, respectively, as shown in Table 3. Precision goals set out by NCEP recommend that HDL-C should be determined with CV < 4% at 42 mg/dL or higher and standard deviation (SD) < 1.7 mg/dL at lower concentrations than 42 mg/dL HDL-C [36]. Therefore, all HDL-MIP QCM results are appreciably within the target criteria. Actually, clinical standard homogeneous assays typically have CV < 1.8–3.1% [7]. In contrast to this, laboratory-based methods, such as enzyme assays or immunoassays, are generally very precise. However, they are usually slower than the assays mentioned above.

**Table 3:** Repeatability test

HDL-C (mg/dL)	Mean (mg/dL) (*n* = 3)	SD	%CV
20	20.19	1.55	7.7
50	53.20	1.23	2.3
100	101.89	3.43	3.4

#### Selectivity

**Fig. 7:**
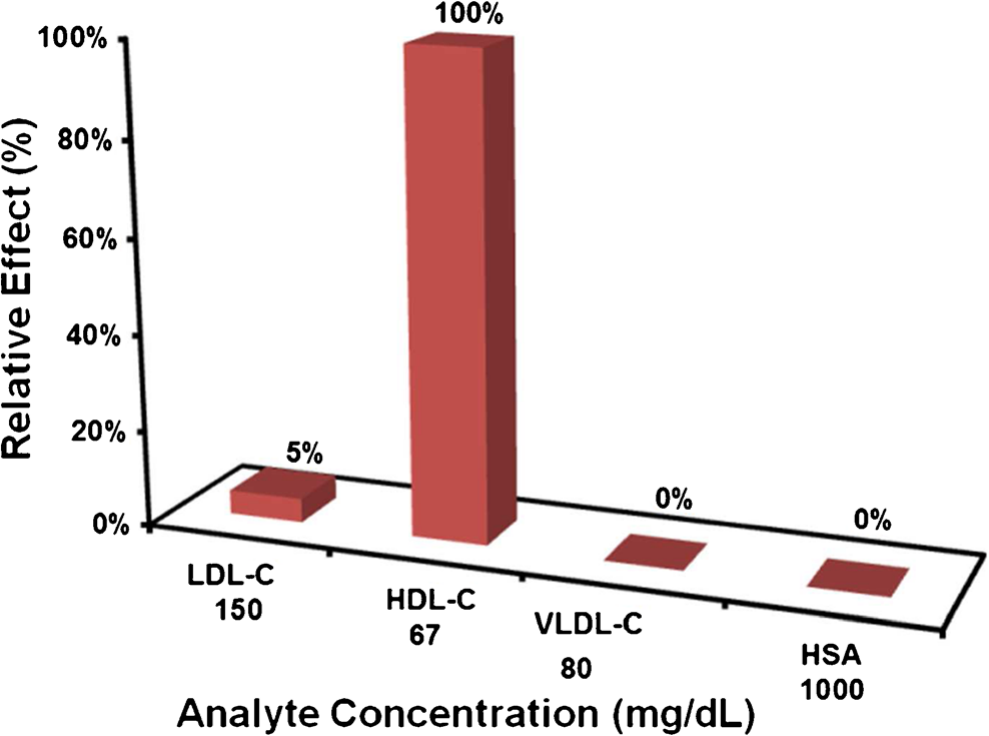
Selectivity of HDL-MIP sensor

Figure 7 summarizes the HDL-MIP sensor responses toward “high” concentrations of HDL-C at 66.66 mg/dL, LDL-C at 150 mg/dL, VLDL-C 80 mg/dL, and HSA at 1000 mg/dL. All these compounds are present in human serum; “high” in this case refers to clinically high concentrations for the respective parameter. The sensor response for HDL-C is up to a factor of 15 or higher, than for all the other compounds despite HDL-C concentrations being the lowest. This means that selectivity of the HDL-MIP for HDL is considerably large. Not considering this difference in concentration, LDL leads to 5% of the HDL signal and VLDL and HSA to 0%, which indicates specificity of HDL-MIP against these (lipo)proteins. As per the previous discussion, there are cavities in the HDL-MIP whose diameter is somewhat larger than the physiological diameter of HDL (21.5 ± 6.5 nm). This explains why some LDL (28.9 ± 9.2 nm) can bind to the cavities and cause a sensor signal. In contrast to that, VLDL particles (30–80 nm in diameter range) are found mostly in the average size of 48.8 nm [37], which is much larger than the diameter of the HDL-MIP cavities. Finally, HSA forms an ellipsoid shape in a diameter of 36 nm [38], which does not properly correspond to the oblate spheroid structure of HDL-MIP cavities. Additionally, the nonimprinted surface reveals low nonspecific binding, indicating a high selectivity of the HDL-MIP.

### Validation of HDL-MIP sensor data

**Fig. 8:**
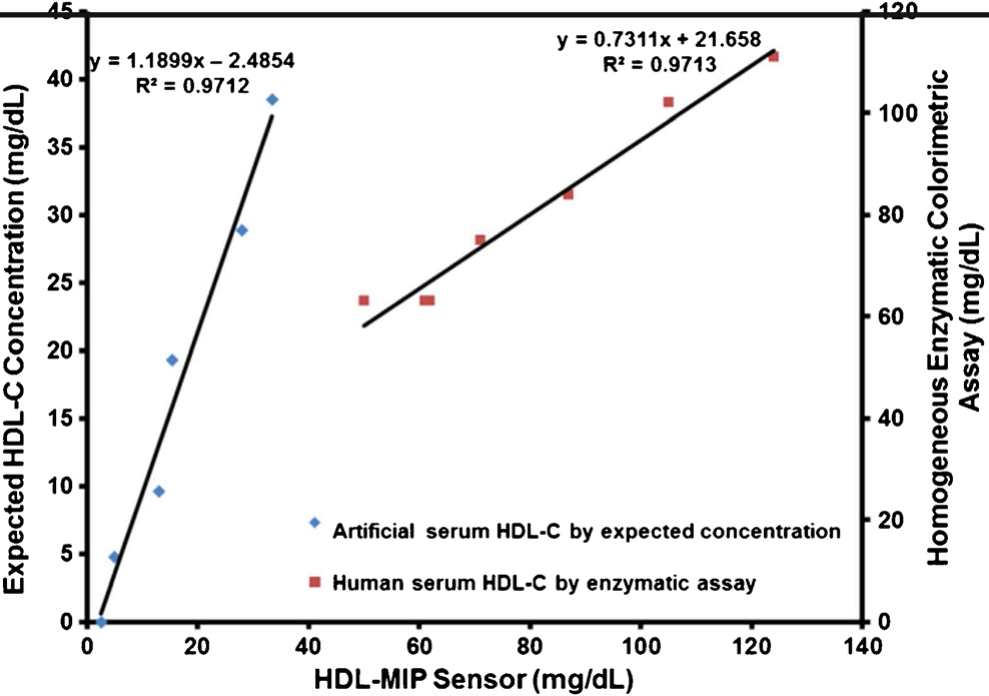
Comparison of HDL-MIP sensor data (*x*-axis) to the expected concentrations of spiked artificial sera (primary *y*-axis) and the enzymatic assay results of the actual sera (secondary *y*-axis)

**Fig. 9:**
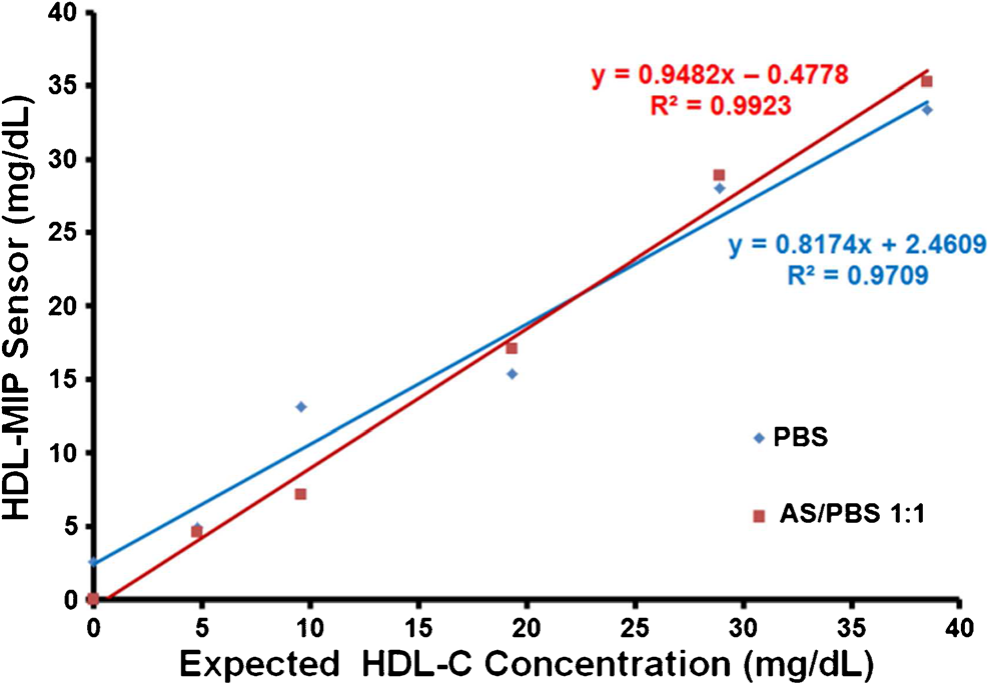
Sensor characteristics of HDL-MIP sensor in both PBS and AS/PBS at 1:1 as running solutions, respectively

Figure 8 combines two different datasets for demonstrating validity of the HDL-MIP sensor approach. The right-hand linear compares the sensor data obtained in human serum with the results of the enzymatic colorimetric standard assay. Obviously, the data of the two methods are well correlated to each other: the correlation coefficient is *R* ^2^ = 0.9713 for the concentration range above 40 mg/dL HDL-C. Hence, the QCM lead to the same results as the clinical standard method, but do so in a much shorter time and without the need of pretreating the sample to isolate HDL. Due to the lack of suitable standards, these tests could not be carried out for lower concentrations. For those samples, we hence carried out intra-assay tests by comparing the sensor responses obtained in artificial serum (as described previously) with the corresponding data of the sensor characteristic. This turned out necessary, because it was not possible to carry out enzymatic assays in the artificial matrix. Again, predicted and measured data correspond very well to each other. The difference in slopes for the two concentration ranges partly results from the different scales of the two vertical axes. The actual difference is 1.19 vs. 0.73 for intra-assay tests and enzymatic colorimetric standard assay, respectively. Obviously, the sensor assay somewhat underestimates low HDL concentrations, which in terms of risk assessment is no problem. The apparent underestimation during validation may be caused by the large intercept of the sensor characteristic and will require further research. However, the same sample containing 0 mg/dL HDL-C generated a small signal at a frequency shift of −45 Hz. This indicates that the difference in viscosity of the assay matrix may lead to slightly overestimating very low HDL-C concentrations. However, Fig. 9 also shows that sensor characteristics are similar in PBS buffer and sera in a way that the corresponding regression parameters *R* ^2^ are similar, namely, 0.9709 in PBS and 0.9923 in AS/PBS, respectively. As in the case of LDL-MIP sensor measurements [24], HDL-MIP sensor measurements required to dilute human serum samples 1:1 with PBS to reduce matrix effects. This does not lead to systematic errors, as the two sensor characteristics in diluted serum and PBS buffer shown in Fig. 9 reveal: they are basically identical.

## Conclusions

The HDL-MIP-based QCM sensor presented here is able to selectively detect HDL in the clinically relevant concentration range, both in (diluted) serum and in buffers. In contrast to existing clinical standard techniques for determining HDL, this sensor does not require any sample pretreatment other than diluting it. It hence represents a reagentless sensing technology and leads to reduced assay complexity and time of measurement. Furthermore, the signals are inherently reversible, making the system potentially useful for long-term measurements in clinical monitoring.
